# From mid-career professor to chairperson: What remains similar what is different?

**DOI:** 10.1192/j.eurpsy.2021.128

**Published:** 2021-08-13

**Authors:** P. Falkai

**Affiliations:** Department Of Psychiatry And Psychotherapy, Univeristy of Munich, Munich, Germany

**Keywords:** Mid-career, Professor, Chairperson, Responsibilities

## Abstract

For a Mid-career Professor in Germany, there are defined clinical and teaching responsibilities. One can focus either on one’s research or on clinical work and teaching. When tasks are becoming more demanding or significant overarching decisions need to be taken, there is always a chairperson to be asked or to help delegate tasks. As chairperson, one is mostly independent from other persons except for the dean of the medical faculty. One is however, at least in Germany, the chairpersons fully responsible for keeping up teaching, patient care, research as well as running the department. The Chairperson is measured by the achievements of these four tasks. It need special attention to keep up a balanced time schedule to cover clinical care, research, teaching and departmental management. A good chairs means working together with your staff on long-term goals, developing the department fruitfully and trying to fulfil these goals.

**Disclosure:**

No significant relationships.

